# Supplementary material for: Embryonic hematopoiesis modulates the inflammatory response and larval hematopoiesis in Drosophila
Source: eLife. 2018 Jul 11;7:e34890. doi: 10.7554/eLife.34890 (PMC6040882; doi:10.7554/eLife.34890)
Supplement: Supplementary file 1 [file elife-34890-supp1.docx]

# Supplementary materials

## Supplementary methods:

### Fly strains and genetics

The following stocks were used:

| Genotypes | Abbreviation | Origin | Remarks |
| --- | --- | --- | --- |
| *w^1118^* | WT | Bloomington #5905 |  |
| *hop^Tum-l^/FM7c* | *hop^Tum-l^* | Bloomington #8492 | point mutation that constitutively activates the Jak/Stat pathway |
| *UAS-hop^Tum-l^/CyO,twilacZ* | *UAS-hop^Tum-l^* | ^1^ | reporter line for *hop^Tum-l^* over-expression |
| *gcmGal4,UAS-mCD8GFP/CyO,Tb* | *gcm>GFP* | ^2^ | driver specific to embryonic hemocytes and glia, *gcm* hypomorphic mutation |
| *UAS-gcmRNAi* | *gcm KD* | Bloomington #31519 | dsRNA reporter line for *gcm* down-regulation |
| *UAS-gcmF18A* | *gcm GOF* | ^3^ | reporter line for *gcm* over-expression |
| *gcm^26^/CyOactinGFP* | *gcm^26^* | ^4^ | null *gcm* mutation |
| *upd2*^Δ^ |  | Bloomington #55727 | 4.7 kb deletion |
| *upd3*^Δ^ |  | Bloomington #55728 | imprecise excision |
| *UAS-upd2RNAi* | *upd2 KD* | Bloomington #33988 | dsRNA reporter line for *upd2* down-regulation |
| *UAS-upd3RNAi* | *upd3 KD* | Bloomington #32859 | dsRNA reporter line for *upd3* down-regulation |
| *UAS-upd2/CyO* | *upd2 GOF* | ^5^ | reporter line for *upd2* over-expression |
| *UAS-upd3/CyO* | *upd3 GOF* | ^5^ | reporter line for *upd3* over-expression |
| *UAS-Ptp61FRNAi* | *Ptp61F KD* | Bloomington #32426 | dsRNA reporter line for *Ptp61F* down-regulation |
| *UAS-Socs36ERNAi* | *Socs36E KD* | Bloomington #35036 | dsRNA reporter line for *Socs36E* down-regulation |
| *UAS-Socs44ARNAi* | *Socs44A KD* | Bloomington #42830 | dsRNA reporter line for *Socs44A* down-regulation |
| *UAS-Ptp61Fa/CyO* | *Ptp61Fa GOF*  (cytoplasmic) | ^6^ | reporter line to over-express the cytoplasmic splicing isoform |
| *UAS-Ptp61Fc/TM3* | *Ptp61Fc GOF*  (nuclear) | ^6^ | reporter line to over-express the nuclear splicing isoform |
| *gcmGal4,UAS-mCD8GFP,repoGal80/CyO* | *gcm(hemo)>* | ^7^ | *gcm* driver not expressed in glia, hypomorphic mutation |
| *snGal4* |  | ^8^ | *singed* driver, specific to embryonic hemocytes |
| *srp(hemo)Gal4* | *srp(hemo)>* | ^9^ | *serpent* driver specific to embryonic hemocytes |
| *DotGal4* |  | Bloomington #67608 | *Dorothy* driver specifically expressed in embryonic and larval lymph gland |
| *lzGal4,UAS-mCD8GFP* | *lz>GFP* | Bloomington #6314 | *lozenge* driver expressed in crystal cells |
| *10xStat92E-GFP* |  | Bloomington #26198 | reporter line for STAT activity, 10 Stat92E binding sites driving GFP expression |
| *UAS-FLP;;Ubi-p63E(FRT.STOP)Stinger* | *gtrace* | Bloomington #28282 | This line allows the analysis of lineage-traced expression of Gal4 drivers |
| *UbiGal4* | *Ubi>* | Bloomington #32551 | Expresses Gal4 in all cells |
| *UAS-RFP* | *RFP* | Bloomington #30556 | Inserted on the 2^nd^ chromosome. |
| *HmlΔRFP* | *HmlΔRFP* | ^10^ | Inserted on the 2^nd^ chromosome. |
| *Act5C(FRT.polyA)lacZ* | *gtrace-LacZ* | Bloomington #6355 |  |

**See also Fig.2-source data 2**

The crosses performed for each experiment are detailed below.

**Fig. 1b**: to assess the impact of Gcm on melanotic tumor formation we used *gcmGal4,UAS-mCD8GFP/CyO;UAS-gcmRNAi* (*gcm>gcm* *KD*) animals that were crossed with *hop^Tum-l^* females (column 4). Rescue experiments of the *hop^Tum-l^* phenotype were performed by over-expressing *gcm* (*gcm* *GOF*) (columns 5 and 6). In details, we crossed *hopTum-l;UAS-gcm* females with *gcmGal4,UAS8mCD8GFP/CyO,Tb* males to obtain *hopTum-l/+;gcmGal4/+;UAS-gcm/+* larvae. We crossed *hopTum-l;UAS-gcm* females with *gcmGal4,UAS8mCD8GFP/CyO;UAS-gcmRNAi* (*gcm>gcm KD*) males to obtain *hopTum-l/+;gcmGal4/+;UAS-gcm/UAS-gcmRNAi* larvae.

**Fig. 2f**: to assess the levels of expression of the Jak/Stat inhibitors in embryos, *gcm KD*, *gcmGal4,UAS-mCD8GFP/CyO,Tb;UAS-gcmRNAi* females were crossed with *UAS-gcmRNAi* males. For the control, *gcmGal4,UAS-mCD8GFP/CyO,Tb* were crossed with *w^1118^*. GFP positive cells from overnight lay were sorted by FACS before assessing the levels of the inhibitors by qPCR. AT least 50 embryos were collected per replicate.

**Fig. 2g**: to assess the impact of Jak/Stat inhibitors on melanotic tumor formation, *Ptp61F*, *Socs36E* and *Socs44A* were silenced using *gcmGal4* in *hop^Tum-l^* mutant animals (columns 2, 3 and 4). *hop^Tum-l^*/+ animals were generated by crossing *hop^Tum-l^* homozygous females with *w^1118^* males (column 1). Rescue experiments of the *hop^Tum-l^/+;gcm>gcm KD* phenotype were performed by over-expressing *Ptp61F* (*Ptp61F* *GOF*). Two splicing isoforms of the carboxyl terminal of the Ptp61F protein were used, the cytoplasmic isoform (*Ptp61Fa* *GOF*) and the nuclear isoform (*Ptp61Fc* *GOF*) ^11^ (columns 6 and 7).

**Fig. 2h**: to assess the levels of expression of Jak/Stat downstream targets in st. 16 embryonic hemocytes, *srp(hemo)Gal4;UAS-RFP* females were crossed with *w^1118^* males (Control) or *UAS-gcmF18A* males. Egg laying were carried out for 3 h, then incubated 12 h at 25°C before FACS sorting according to the RFP signal.

**Fig. 4h**: rescue experiments of the *hop^Tum-l^* phenotype were performed by silencing *upd2* or *upd3* using *gcmGal4* in *hop^Tum-l^* mutant animals. To be consistent with the data in **Fig. 4 – Fig. Supp. 1a**, we analyzed only female larvae.

**Fig. 5, Fig. 5 – Fig. Supp. 1b-f**: *UAS-hop^Tum-l^/CyOactinGFP* females were crossed with *gcmGal4,UAS-mCD8GFP/CyO,Tb*. To assess the role of *gcm, UAS-hop^Tum-l^/CyOactinGFP* females were crossed with *gcmGal4/CyO;UAS-gcmRNAi/+* males, however, the viability of *gcmGal4/UAS-hop^Tum-l^;UAS-gcmRNAi/+* larvae, which all show tumors (100% penetrance), is very low (n=13). To avoid the issue of embryonic lethality generated by the wide expression of the driver, *UAS-hop^Tum-l^/CyOactinGFP* females were crossed with *gcm(hemo)Gal4,UAS-mCD8GFP/CyO,Tb;UAS-gcmRNAi* males to generate or *gcm(hemo)Gal4/UAS-hop^Tum-l^;UAS-gcmRNAi/+* animals. These larvae still show a significantly higher tumor penetrance than that observed in *gcm(hemo)Gal4/UAS-hop^Tum-l^* animals (45.3%), see **Fig. 5 – Fig. Supp. 1a**.

**Fig. 6:**  *srp(hemo)>*, *srp(hemo)>hop^Tum-l^* and *srp(hemo)>hop^Tum-l^, gcm KD* were obtained by crossing *srp(hemo)Gal4* males with *w^1118^* females, *srp(hemo)Gal4* males with *UAS-hop^Tum-l^/CyOactinGFP* females and *srp(hemo)Gal4/CyOactinGFP;UAS-gcmRNAi/TM6,Tb^1^,Hu* with *UAS-hop^Tum-l^/CyOactinGFP* females, respectively.

**Fig. 7**: to assess the impact of *gcm* *KD* on wasp encapsulation, we crossed *gcmGal4* or *UAS-gcmRNAi* or *gcmGal4,UAS-mCD8GFP/CyO,Tb;UAS-gcmRNAi* animals with *w^1118^* to generate *gcmGal4/+* or *UAS-gcmRNAi/+* or *gcmGal4,UAS-mCD8GFP/+;UAS-gcmRNAi/+* respectively.

**Fig. 8 and Fig. 4 – Fig. Supp. 1b,b’**: to sort *gcm^26^* homozygous hemocytes, the following stocks, *srp(hemo)Gal4,gcm^26^/CyOactinGFP* and *UAS-RFP,gcm^26^/CyOactinGFP*, were built by recombining *srp(hemo)Gal4* and *UAS-RFP* with *gcm^26^*. Then, *srp(hemo)Gal4,gcm^26^/CyOactinGFP* was crossed with *UAS-RFP,gcm^26^/CyOactinGFP* to produce homozygous *gcm^26^* embryonic hemocytes that are RFP positive and can be sorted by FACS. For the control, *srp(hemo)Gal4* was crossed with *UAS-RFP*. The hemocytes were sorted from stage 16 embryos.

**Fig. 9d,e:** to track the lineage expressing *gcmGal4* in the immune system, first a *UAS-FLP;gcmGal4/CyOactinGFP* strain was build using the *UAS-FLP* from the *gtrace* flies (B#28282). Then, females *UAS-FLP;gcmGal4/CyOactinGFP* were crossed with males *gtrace-LacZ*, egg laying were carried out for 6 h, then 48 h later, the larvae were infested with wasps.

**Fig. S2a-e**: to assess the impact of the *gcm* mutation on crystal cells’ formation in embryos, *lzGal4,UAS-mCD8GFP* females were first crossed with males *gcm^26^,UAS-RFP/CyOactinGFP*. Males *lzGal4,UAS-mCD8GFP;gcm^26^,UAS-RFP/+* were then crossed with females *gcm^26^/CyOactinGFP*.

**Fig. 1 – Fig. Supp. 3b**: to assess the RNAi efficiency, males homozygous *Ubi>* were crossed with females *UAS-gcmRNAi*. For the control, *UAS-gcmRNAi* females were crossed with *w^1118^* males. Overnight lay were used for assessing *gcm* expression levels by qPCR on at least 50 embryos per replicate.

**Fig. 1 – Fig. Supp. 3h**: to check the impact of the *gcm* mutation on crystal cells’ formation in larvae, *lzGal4,UAS-mCD8GFP* females were crossed with males of one of the following genotypes: *w^1118^*, the null mutation *gcm^26^*, *gcmGal4,UAS-mCD8GFP/CyO,Tb* or *gcmGal4,UAS-mCD8GFP/CyO;UAS-gcmRNAi* (*gcm>gcm* *KD*).

**Fig. 1 – Fig. Supp. 4a-c’**: to check the impact of the *gcm* mutation on lamellocyte formation, *gcmGal4,UAS-mCD8GFP/CyO,Tb;UAS-gcmRNAi* females were crossed with *UAS-gcmRNAi* males and *gcmGal4,UAS-mCD8GFP/CyO,Tb* females were crossed with *w^1118^* males. Both crosses were set at 25°C for 24h. The tubes containing embryos were then shifted to 29°C until 3^rd^ instar larval stage to enhance the phenotype.

**Fig. 1 – Fig. Supp. 4e**: to assess the impact of Gcm on melanotic tumor formation we used the null mutation *gcm^26^* animals that was crossed with *hop^Tum-l^* females (column 2). To confirm that the observed phenotypes arise from defects in the hemocytes, *hop^Tum-l^* females were crossed with *gcm(hemo)Gal4* that induces transcription in embryonic hemocytes but not in glia, the other main territory of Gcm expression (columns 3 and 4) or with embryonic hemocyte drivers *srp(hemo)Gal4* and *snGal4* (columns 5, 6, 7, 8, 9 and 10).

**Fig. 4 – Fig. Supp. 1a**: to assess melanotic tumor penetrance in double mutant animals, we only analyzed female larvae, as *hop*, *upd2* and *upd3* are all located on the 1^st^ chromosome.

**Fig. 5 – Fig. Supp. 1a:** we crossed *srp(hemo)Gal4*, *snGal4* and *gcm(hemo)Gal4* animals with *UAS-hop^Tum-l^/CyOactinGFP* animals to confirm the phenotype obtained using the *gcm* driver.

### List of primers

| Species | Gene | Forward | Reverse |
| --- | --- | --- | --- |
| Drosophila | *Gapdh1* | CCCAATGTCTCCGTTGTGGA | TGGGTGTCGCTGAAGAAGTC |
| Drosophila | *Act5c* | GCCAGCAGTCGTCTAATCCA | GACCATCACACCCTGGTGAC |
| Drosophila | *Ptp61F* | GAAACTGCCCCACGTCAAAC | CTTAAGGAATGCGTTCGGCG |
| Drosophila | *Socs36E* | GTGTCCAACACCAGCTACGA | GAGACCCGTATGTTGACCCC |
| Drosophila | *Socs44A* | CACTCCAAAATGAGCCACGG | GAGTGGAACCAGCCCTTCTT |
| Drosophila | *upd2* | ACCCTGGAGTACGGCAATCT | CTGATCCTTGCGGAACTTGT |
| Drosophila | *upd3* | CCACAGTGAGCACCAAGACT | CAGGTCCCAGTGCAACTTGA |
| Drosophila | *crq* | gcgatcatcgaagcgggaag | gcattagcttctgatggctc |
| Drosophila | *Hml* | ccgatgatgacgacgaggat | gatgttgaagctaatgtggc |
| Drosophila | *lectin-24A* | CAATGCCTACAGCCAGGATT | AGGCTAGGTGACCTCCCATT |
| Drosophila | *eater* | CGTCTGTCAATGCCTGACGG | AGACACCTTCCAGCTTCGTG |
| Drosophila | *He* | GGCGGAGCAGTTCACACTAA | AGTTGGAGATGGACGGTTGC |
| Drosophila | *NimC1* | TCCAATGCCTTTGGGTGTGT | GGTGCGGTATTTTGTCTGCC |
| Drosophila | *Filamin-240 (cher)* | CGGATCAGTACGAGGAGAAC | GATCGATGGTCTTCAGGTGC |
| Drosophila | *α-PS5 (ItgaPS5)* | ACTTCGGTTACTCCGTGGTG | GCACCCACGTCATAGGAATC |
| Drosophila | *mys* | GATCACGGTACATGCGAGTG | GTACCATGACCGGAGCAGAT |
| Drosophila | *βInt-v (Itgbn)* | CTCGCCGGCAACTACTTAAC | GGACAGCCTGATCACTGGTT |
| Drosophila | *Tep1* | ctgaagtctcagtcagcctgactggacctt | CGTAATCGCCTTCTGTTAGCTTCGGAATGT |
| Drosophila | *Tep4* | GTCAATGTCCATCTGGACTC | GAAGTCCTTGAGATCCATGG |
| Drosophila | *PPO3* | AGAGCGTGGCGGTGTACGCCAGGGATCGCG | CTTGGGGAAGTAGCCCTCGGCAATTGGTTC |
| Drosophila | *lz* | CTCCAACTCCATCAGCATCT | CCAATCCGAGTCCGAGTCCG |
| Drosophila | *hnt (peb)* | TTTCAACGGGAACCAAGCCT | AGCATTTTTCCAACGGCTAGTT |
| Drosophila | *PPO1* | GATACTCGCGCGCTACAATG | GGTTATTCGTGCTGGACAGG |
| Drosophila | *apt* | TCGATCTGTGTCGCAAGGAC | TCGTTGCGAGTACATGCCTT |
| Drosophila | *CG1572* | TCATGCGAAGCAGGAGAAGG | GCGCCAAAGAACGTTTCACA |
| Drosophila | *CG13559* | TCGGGCATAGTCTTGTTCACC | CGGAACGATTCCCAGGGTC |
| Drosophila | *Galphaf* | CTGCGACCACGTCACTACTT | CATCGCTGATCCGCACAAAG |
| Drosophila | *slbo* | TGGTGAGATGACCAACGAGC | TCGCAGGACACTGGCTTTAG |

## Supplementary Figures

**Fig. 1 – Fig. Supp. 1. Gcm is not expressed in the second hematopoietic wave.** Control lineage tracing in the lymph gland of Wandering L3 using the lymph gland specific *Dot>gtrace* line ^12^ (**a,a’**). Lineage tracing in Wandering L3 using *gcm>gtrace* (**b,b’**) and *hop^Tum-l^;gcm>gtrace* lines (**c,c’**). The gtrace construct allows the constitutive expression of GFP as soon as the driver (here *Dot>* or *gcm>*) is expressed in the cell. Thus, the GFP signal indicates cells that have expressed the driver during development and/or are still expressing it ^13^. DAPI in blue and gtrace in white, maximum Z-projections. Note the expression of Dot in all the cells of the lymph gland (**a,a’**) and the absence of Gcm expression (**b,b’**) even upon constitutive Jak/Stat activation (**c,c’**). (**d-d’’’**) Control lineage tracing showing Gcm expression in the larval nervous system. In this case, the *gcm>gtrace,UAS-RFP* construct makes it possible to specifically identify the cells currently expressing Gcm as RFP positive. Gcm is expressed in many cell lineages (glia for gtrace signal and lamina neurons^14^ for RFP + gtrace signals).

**Fig. 1 – Fig. Supp. 2. Crystal cell phenotype in *gcm^26^* embryos.** (**a-b’**) Immunolabelling of *gcm^26^/CyOactinGFP* (**a,a’**) and *gcm^26^* homozygous embryos (**b,b’**) (DAPI in blue, GFP in green, PPO1 crystal cell marker in red). (**a,b**) show merge of the three channels and (**a’,b’**) show PPO1 alone. (**c-d’**) Immunolabelling of *lz>RFP,gcm^26^/CyOactinGFP* (**c,c’**) and *lz>RFP,gcm^26^* embryos (**d,d’**) (DAPI in blue, GFP in green, RFP in red). (**c,d**) show merge of the three channels and (**c’,d’**) show RFP labelling alone. (**e**) Number of crystal cells counted in stage 13 *lz>RFP,gcm^26^/CyOactinGFP* and *lz>RFP,gcm^26^* embryos. Note that in the mutant background, crystal cell labelling is also observed at ectopic positions, scattered along the embryo (white arrowheads in **b’** and **d’**) and the total number of crystal cells increases compared to that observed in heterozygous embryos, in agreement with previous data ^15^.

**Fig. 1 – Fig. Supp. 3. Crystal cell phenotype in *gcm KD* larvae.** (**a**) *gcm RNAi* efficiency in S2 cells detected by GFP intensity in *gcm GOF* + *gcm KD* transfected S2 cells as compared to controls (n=9). GFP signal measured upon transfection with *pPac-gal4*, *pUAS-gcm* and *repo-GFP* plasmids (column 2) or *pPac-gal4*, *pUAS-gcm*, *pUAS-gcmRNAi* and *repo-GFP* plasmids (column three) respectively. *repo-GFP* represents the reporter for Gcm activity ^16^. (**b**) *gcm RNAi* efficiency in embryos. The levels of *gcm* were measured by qPCR on embryos (overnight lay) of the following genotypes: *UAS-gcmRNAi/+* (Control) and *Ubi>gcm KD* (*Ubi>* is a driver ubiquitously expressed). The levels are represented relative to the two housekeeping genes *Gapdh1* and *Act5c*. (**c**) Relative expression levels of crystal cell markers in hemocytes from *gcm>* and *gcm>gcm KD* Wandering L3 measured by qPCR and normalized to two housekeeping genes (n=4). (**d-f’**) Crystal cells visualized in Wandering L3 after heat treatment at 70°C for 10 min. (**g**) Average number of crystal cells/larva in *WT*, *gcm>* and *gcm>gcm KD* animals (n=6). (**h**) Average percentage of crystal cells in circulating and sessile compartments in the mentioned genotypes, using the *lz>GFP* driver specific to crystal cells ^17,18^ (n=3).

**Fig. 1 – Fig. Supp. 4. Gcm inhibits Jak/Stat-mediated melanotic tumor formation.** (**a-c**) Immunolabelling of hemocytes from Wandering L3 of the mentioned genotypes (DAPI in blue, Phalloidin in green, Srp in red, lamellocyte marker L4 in white), (n>3). Strong Phalloidin labelling is specific to lamellocytes ^19,20^. In all fluorescent confocal images, maximum Z-projections are presented. (**d**) Melanotic tumors of different size (arrowheads). See material and methods for quantitative assessment. (**e**) Tumor penetrance in *hop^Tum-l^*/+*;gcm^26^/+* larvae (column 2), *hop^Tum-l^*/+*;gcm(hemo)Gal4/+* (column 3), *hop^Tum-l^*/+*;srp(hemo)Gal4/+* (column 6) and *hop^Tum-l^*/+*;snGal4*/+ (column 9) or upon *gcm KD* (columns 4, 7 and10) as compared to controls (n>50). (**f**) Percentage of S2 cells presenting nuclear Stat92E labelling after transfection with *pPac-gal4* and *pUAS-RFP* (Control), or *pPac-gal4*, *pUAS-RFP* and *pUAS- hop^Tum-l^* (*hop^Tum-l^* *GOF*), or *pPac-gal4*, *pUAS-RFP*, *pUAS- hop^Tum-l^* and *pPac-gcm* (*hop^Tum-l^* *GOF, gcm GOF*). The quantification was done on at least 20 cells in triplicates. The labelling is displayed in **Fig. 2a-c**. (**g-i**) Loci containing DamID peaks (black), Gcm binding sites (GBSs, in red), blue arrows within the loci indicate the direction of transcription, histograms above the locus show a region of 1kb on each side of a DamID peak scoring a FDR < 0.001, genomic coordinates of the loci are indicated above the histograms: *Socs44A* (**g**), *Ptp61Fa* (cytoplasmic isoform) and *Ptp61Fc* (nuclear isoform) obtained upon alternative splicing at the 3’ carboxyl terminal of *Ptp61F* (**h**) and *Socs36E* (**i**).

**Fig. 4 – Fig. Supp. 1. Interaction between Jak/Stat pathway, Gcm and Upd2/Upd3 cytokines.** (**a**) Tumor penetrance in double heterozygous female larvae *hop^Tum-l^/upd2^Δ^* and *hop^Tum-l^/upd3^Δ^*. (**b,b’**) *upd2* and *upd3* expression levels in hemocytes sorted from stage 16 control embryos (*srp(hemo)Gal4/UAS-RFP*) or *gcm^26^* embryos (*srp(hemo)Gal4, gcm^26^/UAS-RFP,gcm^26^*). Note that the levels of *upd2* and *upd3* are at the limit of the detection range in WT hemocytes and their levels increase strongly in *gcm^26^* hemocytes. (**c,d**) Canonical Stat92E binding sites (TTC(N)_3-4_GAA) ^21^ at *upd2* and *upd3* loci (in red), symbols as in **Fig. 1 – Fig. Supp. 1**. (**e**) Total number of circulating hemocytes in the indicated genotypes (n=3).

**Fig. 5 – Fig. Supp. 1. Phenotypes induced by conditional activation of the Jak/Stat pathway in the embryonic hemocytes.** (**a**) Tumor penetrance in conditional *hop^Tum-l^* animals (*UAS-hop^Tum-l^*) using *srp(hemo)>*, *sn>*, *gcm>* and *gcm(hemo)>* drivers as compared to *hop^Tum-l^*/+ (n>50). (**b**) Tumor expressivity assessed as tumor size (n=40). (**c**) Fraction of PH3 positive (dividing) cells in circulating and sessile compartment in the systemic and conditional *hop^Tum-l^* mutations as compared to controls (n=3). (**d-f**) Percentage of lamellocytes in whole hemocyte population (circulating + sessile, Total) (**d**), circulating (**e**) and sessile (**f**) compartments in the mentioned genotypes (n=3). (**g-i**) *10XStat92E-GFP* reporter intensity in somatic muscles. (**j-k**) Relative expression levels of *upd2* (**j**) and *upd3* (**k**) in hemocytes from wandering L3 hemocytes *srp(hemo)>* and *srp(hemo)>hop^tum-1^*, n=3.

**Fig. 8 – Fig. Supp. 1. Gcm is not induced in circulating hemocytes nor in lymph glands of 3^rd^ instar larvae upon wasp infestation.** Immunolabelling of hemocytes from 3^rd^ instar larvae *gcm>GFP* without (**a**) or after wasp infestation (**b**). (DAPI in blue, GFP in green and Phalloidin in gray). (**c**) Immunolabelling of the lymph gland from 3^rd^ instar larva *gcm>GFP* after wasp infestation. (DAPI in blue, GFP in green). (**d**) Bright-field image of the melanised tumor induced by wasp infestation in *gcm>gtrace-LacZ* Wandering L3 shown in **Fig. 9e**. Scale bar: 100 µm.

## Supplementary Bibliography

1 Harrison, D. A., Binari, R., Nahreini, T. S., Gilman, M. & Perrimon, N. Activation of a Drosophila Janus kinase (JAK) causes hematopoietic neoplasia and developmental defects. *The EMBO journal* **14**, 2857-2865 (1995).

2 Soustelle, L. & Giangrande, A. Novel gcm-dependent lineages in the postembryonic nervous system of Drosophila melanogaster. *Developmental dynamics : an official publication of the American Association of Anatomists* **236**, 2101-2108, doi:10.1002/dvdy.21232 (2007).

3 Bernardoni, R., Vivancos, V. & Giangrande, A. glide/gcm is expressed and required in the scavenger cell lineage. *Developmental biology* **191**, 118-130 (1997).

4 Vincent, S., Vonesch, J. L. & Giangrande, A. Glide directs glial fate commitment and cell fate switch between neurones and glia. *Development* **122**, 131-139 (1996).

5 Jiang, H. *et al.* Cytokine/Jak/Stat signaling mediates regeneration and homeostasis in the Drosophila midgut. *Cell* **137**, 1343-1355, doi:10.1016/j.cell.2009.05.014 (2009).

6 Muller, P., Kuttenkeuler, D., Gesellchen, V., Zeidler, M. P. & Boutros, M. Identification of JAK/STAT signalling components by genome-wide RNA interference. *Nature* **436**, 871-875, doi:10.1038/nature03869 (2005).

7 Cattenoz, P. B. *et al.* Functional Conservation of the Glide/Gcm Regulatory Network Controlling Glia, Hemocyte, and Tendon Cell Differentiation in Drosophila. *Genetics* **202**, 191-219, doi:10.1534/genetics.115.182154 (2016).

8 Zanet, J. *et al.* Fascin promotes filopodia formation independent of its role in actin bundling. *The Journal of cell biology* **197**, 477-486, doi:10.1083/jcb.201110135 (2012).

9 Bruckner, K. *et al.* The PDGF/VEGF receptor controls blood cell survival in Drosophila. *Developmental cell* **7**, 73-84, doi:10.1016/j.devcel.2004.06.007 (2004).

10 Makhijani, K., Alexander, B., Tanaka, T., Rulifson, E. & Bruckner, K. The peripheral nervous system supports blood cell homing and survival in the Drosophila larva. *Development* **138**, 5379-5391, doi:10.1242/dev.067322 (2011).

11 McLaughlin, S. & Dixon, J. E. Alternative splicing gives rise to a nuclear protein tyrosine phosphatase in Drosophila. *J Biol Chem* **268**, 6839-6842 (1993).

12 Kimbrell, D. A., Hice, C., Bolduc, C., Kleinhesselink, K. & Beckingham, K. The Dorothy enhancer has Tinman binding sites and drives hopscotch-induced tumor formation. *Genesis* **34**, 23-28, doi:10.1002/gene.10134 (2002).

13 Evans, C. J. *et al.* G-TRACE: rapid Gal4-based cell lineage analysis in Drosophila. *Nature methods* **6**, 603-605, doi:10.1038/nmeth.1356 (2009).

14 Chotard, C., Leung, W. & Salecker, I. glial cells missing and gcm2 cell autonomously regulate both glial and neuronal development in the visual system of Drosophila. *Neuron* **48**, 237-251, doi:10.1016/j.neuron.2005.09.019 (2005).

15 Bataille, L., Auge, B., Ferjoux, G., Haenlin, M. & Waltzer, L. Resolving embryonic blood cell fate choice in Drosophila: interplay of GCM and RUNX factors. *Development* **132**, 4635-4644, doi:10.1242/dev.02034 (2005).

16 Laneve, P. *et al.* The Gcm/Glide molecular and cellular pathway: new actors and new lineages. *Developmental biology* **375**, 65-78, doi:10.1016/j.ydbio.2012.12.014 (2013).

17 Waltzer, L., Ferjoux, G., Bataille, L. & Haenlin, M. Cooperation between the GATA and RUNX factors Serpent and Lozenge during Drosophila hematopoiesis. *The EMBO journal* **22**, 6516-6525, doi:10.1093/emboj/cdg622 (2003).

18 Osman, D. *et al.* A Drosophila model identifies calpains as modulators of the human leukemogenic fusion protein AML1-ETO. *Proc Natl Acad Sci U S A* **106**, 12043-12048, doi:10.1073/pnas.0902449106 (2009).

19 Avet-Rochex, A. *et al.* An in vivo RNA interference screen identifies gene networks controlling Drosophila melanogaster blood cell homeostasis. *BMC Dev Biol* **10**, 65, doi:10.1186/1471-213X-10-65 (2010).

20 Stofanko, M., Kwon, S. Y. & Badenhorst, P. Lineage tracing of lamellocytes demonstrates Drosophila macrophage plasticity. *PloS one* **5**, e14051, doi:10.1371/journal.pone.0014051 (2010).

21 Yan, R., Small, S., Desplan, C., Dearolf, C. R. & Darnell, J. E., Jr. Identification of a Stat gene that functions in Drosophila development. *Cell* **84**, 421-430 (1996).
